# Improved the Activity of Phosphite Dehydrogenase and its Application in Plant Biotechnology

**DOI:** 10.3389/fbioe.2021.764188

**Published:** 2021-11-25

**Authors:** Tongtong Liu, Lili Yuan, Suren Deng, Xiangxian Zhang, Hongmei Cai, Guangda Ding, Fangsen Xu, Lei Shi, Gaobing Wu, Chuang Wang

**Affiliations:** ^1^ Microelement Research Center, College of Resources and Environment, Huazhong Agricultural University, Wuhan, China; ^2^ Key Laboratory of Arable Land Conservation (Middle and Lower Reaches of Yangtze River), MOA, Huazhong Agricultural University, Wuhan, China; ^3^ Oil Crops Research Institute, Chinese Academy of Agricultural Sciences, Wuhan, China; ^4^ State Key Laboratory of Agricultural Microbiology, College of Plant Science and Technology, Huazhong Agricultural University, Wuhan, China

**Keywords:** phosphite dehydrogenase, phosphite, directed evolution, phosphate, transgenic, fertilizer

## Abstract

Phosphorus (P) is a nonrenewable resource, which is one of the major challenges for sustainable agriculture. Although phosphite (Phi) can be absorbed by the plant cells through the Pi transporters, it cannot be metabolized by plant and unable to use as P fertilizers for crops. However, transgenic plants that overexpressed phosphite dehydrogenase (PtxD) from bacteria can utilize phosphite as the sole P source. In this study, we aimed to improve the catalytic efficiency of PtxD from *Ralstonia* sp.4506 (PtxD_R4506_), by directed evolution. Five mutations were generated by saturation mutagenesis at the 139th site of PtxD _R4506_ and showed higher catalytic efficiency than native PtxD_R4506_. The PtxD_Q_ showed the highest catalytic efficiency (5.83-fold as compared to PtxD_R4506_) contributed by the 41.1% decrease in the *K*_m_ and 2.5-fold increase in the *k*_cat_ values. Overexpression of PtxD_Q_ in *Arabidopsis* and rice showed increased efficiency of phosphite utilization and excellent development when phosphite was used as the primary source of P. High-efficiency PtxD transgenic plant is an essential prerequisite for future agricultural production using phosphite as P fertilizers.

## Introduction

Phosphorus (P) is one of the essential major macronutrients required by all living organisms. It is a limiting nutrient that controls growth in many ecosystems (Du et al., 2020). P in the living systems occurs mainly in the form of inorganic phosphate (Pi) and phosphate esters (C-O-P or P-O-P). The formation and breakdown of phosphate esters under the control of kinases and phosphatases regulates the temporal protein activity and is responsible for the generation, distribution, and utilization of free energy throughout the cell in a number of metabolic pathways (Deng et al., 2020). Moreover, P is a structural component of the phospholipid bilayer membranes and genetic material including DNA and RNA (Matthew et al., 2010). In nature, most P exists in its completely oxidized state (valence of +5) as phosphate anion (PO_4_^3-^, Pi), phosphate-containing minerals, and organic phosphate esters. However, reduced P compounds in the environment may be as high as 10%–20% of total dissolved P in the environment (Benitez-Nelson, 2015). Phosphonates, which contain a carbon-phosphorus (C-P) bond and are characterized by phosphite ester, are found in a wide range of organisms (Andrea and William, 2007).

In general, Pi compounds are the only form of P utilized by the plants for their nutrition. Modern agriculture is currently dependent on the continuous input of Pi fertilizers, produced by the mining of rock Pi (Gilbert, 2009; Achary et al., 2017). Approximately 80% of the mined P is used to manufacture Pi fertilizer (Achary et al., 2017). However, rock Pi is a nonrenewable resource and declining rapidly due to its high rate of consumption (Gilbert, 2009). The rate of the depletion of the Pi rock reserves is accelerated by a low utilization efficiency of the Pi fertilizers in agriculture (Achary et al., 2017). On average, only 20%–30% of applied Pi fertilizers for cultivation are utilized by the plants. The inefficient utilization of Pi fertilizers is due to their high reactivity with soil cations (Fe, Al, and Ca) and rapid conversion into various organic forms by soil microbial flora, which cannot be taken up by plants (Lopez-Arredondo et al., 2014). This also routinely leads to excessive application of P fertilizers in fields, which, in turn, results in increased production costs and environmental degradation due to eutrophication (Shen et al., 2011).

Phosphite (Phi) is a reduced form of Pi with one less oxygen atom (P valence of +3). Phi compounds have been widely used in agriculture as fungicides for controlling several plant diseases caused by Oomycete pathogens including *Phytophthora* spp. (Thao and Yamakawa, 2009; Achary et al., 2017). Although Phi can be absorbed by the plant cells through the Pi transporters, it does not provide P nutrition to the plants (Danova-Alt et al., 2008; Thao and Yamakawa, 2009). The supply of Phi attenuates the Pi starvation responses (PSRs) in plants and inhibits the growth in Pi starved plants (Ticconi et al., 2001; Jost et al., 2015). Several studies show that Phi application has detrimental effects on the growth and development of various plants. Therefore, Phi compounds cannot be used as P fertilizers in agriculture (Thao and Yamakawa, 2009).

Unlike plants, a few bacteria can utilize Phi as the sole source of P. Microorganisms encode phosphite dehydrogenase (PtxD) that can catalyze the oxidization of reduced Phi into Pi with NAD as the cofactor (Andrea and William, 2007). The first *ptxD* gene was cloned and characterized in *Pseudomonas stutzeri* WM88 (Costas et al., 2001). Interestingly, overexpression of the *ptxD*_*PS*_ gene in *Arabidopsis* and tobacco leads to the oxidation of Phi to Pi and consequently enables plants to use Phi as the sole P source for growth and development (López-Arredondo and Herrera-Estrella, 2012). Further studies have successfully applied the *ptxD*_*PS*_/Phi system in rice, maize, and cotton (Manna et al., 2016; Nahampun et al., 2016; Pandeya et al., 2018). In addition to the *ptxD*_*PS*_ gene, the *phoA* from *E. coli* that encodes a bacterial alkaline phosphatase and contains Phi metalizing properties can confer Phi utilization ability in transgenic rice (Ram et al., 2019). PtxD also has displayed great potential in plant biotechnology as it can serve as a novel selectable marker for plant genetic transformation (López-Arredondo and Herrera-Estrella, 2013). In addition, it can be used for the development of phosphite-based novel weed management and fertilization system (López-Arredondo and Herrera-Estrella, 2012; Manna et al., 2016; Pandeya et al., 2018).

The free Pi concentration in the cytosol of plant cells under Pi-sufficient condition is a few tens of micromoles per liter (Mimura, 1999). There is competitive uptake of Phi and Pi through the Pi transporters in the plasma membrane (Danova-Alt et al., 2008). Although the exact concentration of Phi in the cytosol of plant cells is still not measured, the plants can only uptake limited Phi at relatively high concentrations of Pi in the environment (Danova-Alt et al., 2008; Thao and Yamakawa, 2009). This could explain why the overexpression of *ptxD*_*PS*_ only showed a significant effect for Phi utilizing efficiency under no or low Pi (LP) conditions (López-Arredondo and Herrera-Estrella, 2012; Manna et al., 2016; Heuer et al., 2017). Therefore, the *ptxD*_*PS*_ overexpression lines are only suited to grow on lands with very low P content. We believe that PtxD with high affinity and catalytic efficiency will be a superior alternative to PtxDps in plant biotechnology. It has been reported that PtxD from *Ralstonia* sp.4506, referred to as PtxD_R4506_, possesses the highest phosphite affinity among the known PtxD orthologs (Hirota et al., 2012). The better profiles of PtxD_R4506_ have attracted increasing attention in the biotechnological field. Most recently, some properties of PtxD_R4506_, such as cofactor specificity, thermostablity, and organic solvents tolerance, have been evolved by several groups (Liu et al., 2019; Abdel-Hady et al., 2021; Liu et al., 2021). However, to the best of our konwledge, there is no report on the improvement of PtxDR_4506_ catalytic efficiency, which is also extremely important for its application. Unlike PtxDps, PtxDR_4506_ has never been utilized in plant biotechnology.

In the present study, through directed evolution strategy, we have selected several variants of PtxD_R4506_ displaying improved catalytic efficiency toward Phi. Then, the most improved variant (PtxD_Q_) that showed the highest catalytic efficiency (5.83-fold vs. its parent) was then transformed into *Arabidopsis* and rice to evaluate the application potential of PtxD_Q_ encoding gene in plant bioengineering.

## Materials and Methods

### Construction and Screening of Random Mutant Library

A random mutation library of *ptx*D was constructed by error-prone PCR. The PCR mixture (50 μl) was consisted of Taq buffer containing 5 mM MgCl_2_, 20 ng of template plasmid pGEX-6p-*ptx*D, 1 mM dNTPs, 0.2 mM dTTP and dCTP, 0.6 mM MnCl_2_, 2.5 units of Taq DNA polymerase, and 0.4 μM primers (*ptx*D-F and *ptx*D-R). After PCR, the amplified products were purified and double digested with *Bam*HI and *Xho*I. They were then ligated into the expression vector pGEX-6p-1 using T4 DNA ligase. Finally, the ligation product was transformed into *E. coli* DH5*α* and plated onto LB-ampicillin medium to generate the random mutation library.

To screen for the activity-improved variants in the above mutation library, protein induction coupled with T7 phage-mediated cell lysis was performed. Briefly, single colonies from the mutant library were picked and grown in a 96-deep well plate (Qiagen, Germany) containing 600 μl of LB medium with ampicillin (100 mg/ml). After overnight incubation at 37°C, 200 μl of fresh LB culture containing 0.1 mM IPTG and T7 phage was added to each well. This was incubated at 22°C for 6 h at 200 rpm. Subsequently, aliquots for cell lysis (10 μl) were transferred to another 96-well plate with each well containing 150 μl of reaction buffer [50 mM phosphite, 5 mM NAD^+^, and Nitro blue Tetrazolium Chloride (NBT; 0.5 mg/ml)]. After 30 min of incubation at 37°C, the amount of NBT reduction in each well, which corresponded to the activity of the variant, was determined by measuring the absorbance at 550 nm using a Multiskan Spectrum spectrophotometer (Thermo Fisher Scientific, Vantaa, Finland).

### Site-Saturation Mutagenesis

For the most improved variant from the random mutation library, site saturation mutagenesis was performed at position 139Y by overlapping extension PCR (Heckman and Pease, 2007). For each mutagenesis, specific primer pairs containing desired nucleotide mutations (as listed in Supplementary Table S1) were used. The PCR mixture (50 μl) consisted of the PCR buffer, 10 ng of template pGEX-6p-*ptx*D, 0.2 μM pair of flanking primers, and a pair of corresponding specific primers, 0.3 μM dNTPs, and 1 unit of *pfu* DNA polymerase (TransGen, China). After the PCR, the full-length genes containing the desired mutation were ligated into the expression vector pGEX-6p-1. The recombinant vector was transformed into *E. coli* DH5α, and the introduction of the desired mutations was confirmed by sequencing the DNA.

### Protein Expression and Purification

The codon-optimized gene encoding PtxD was chemically synthesized by GenScript Corporation (Naing, China) and inserted into *E. coli* pGEX-6p-1 expression vector, resulting in a recombinant pGEX-6p-PtxD vector. For protein expression, *E. coli* BL21 (DE3) cells with the recombinant plasmid were grown overnight at 37°C in LB broth containing ampicillin (100 μg/ml). Subsequently, the culture was inoculated into fresh LB medium and grown at 37°C till OD600 was 0.6; IPTG (isopropyl-D-thiogalactopyranoside) was then added to induce protein expression at a final concentration of 0.1 mM. After an overnight induction at 18°C, cells were harvested, centrifuged, and washed twice before being resuspended in ice-cold phosphate-buffered solution (140.0 mM NaCl, 2.7 mM KCl, 10.0 mM Na_2_HPO_4_, and 1.8 mM KH_2_PO4; pH 7.0). The suspended solids were disrupted by a French cell press and the lysates were centrifuged at 12,000 g for 30 min. The supernatants were collected to purify the recombinant enzyme by using the GST fusion protein purification kit according to the instructions of the manufacturer. The purity of the recombinant PtxD and its variant enzymes were analyzed using 12% SDS-PAGE (sodium dodecyl sulfate–polyacrylamide gel electrophoresis). The protein concentrations were measured by the Bradford method using the bovine serum albumin standard.

### Enzyme Assay

The kinetic parameters for the wild-type (WT) PtxD and its variants with phosphite were assayed as described previously (Hirota et al., 2012). The enzymatic reaction consisted of 100 mM Tris-HCl buffer containing 5 mM NAD^+^ (saturating concentration), sodium phosphite at varying concentrations (ranging from 0.02 to 2 mM), and the purified enzyme. The reaction was performed at 30°C, and the initial rates were determined by measuring the absorbance of NADH at 340 nm. The kinetic constants were analyzed by the Michaelis–Menten equation using the initial reaction parameters and phosphite concentration in GraphPad Prism 5.0. The effects of pH, temperature, and common ions at the final concentration of 1 mM on the activity of PtxD were assayed *via* routine methods described elsewhere (Li et al., 2020).

### Generation of Transgenic Plants

To generate plants overexpressing *ptxD*_*Q*_, the full-length coding sequence of *ptxD*_*Q*_ was inserted into the pTF101 vector driven by the Ubi promoter at *Bam*HI restriction sites. The full-length coding sequence of *ptxD*_*Q*_ was also inserted into the *pCAMBIA1300* vector driven by the *CaMV 35S* promoter at *Bam*HI restriction sites. The two vectors were constructed using GB clonart Seamless Cloning Kit (GBI, Suzhou, China) according to the instructions of the manufacturer. The sequences of the *ptxD*_*Q*_ primers were listed in Supplementary Table S2.

The *pTF101-ptxD*_*Q*_ was transformed into Agrobacterium tumefaciens strain GV3101 and then transformed into *Arabidopsis* (*Arabidopsis thaliana*) using the floral dip method. All WT and transgenic *Arabidopsis* plants used in this study were in the Columbia background. The *pTF101-ptxD*_*Q*_ was recombined into Agrobacterium tumefaciens strain EHA105, and transgenic rice was generated by Agrobacterium-mediated transformation as described in previous studies (Lu et al., 2016). The resultant transgenic plants were confirmed by testing their resistance to Basta and RT-PCR analysis. Total RNA was extracted from the tissues of rice plants using TRIzol reagent (Thermo Fisher Scientific). Total RNA (2 μg) from each sample was used to synthesize complementary DNA (cDNA) using a High-Capacity cDNA reverse transcription kit (Thermo Fisher Scientific); 30 cycles in RT-PCR were used. The *ACTIN* genes of *Arabidopsis* and rice were used as the internal references, respectively. The sequences of the primers were listed in Supplementary Table S2.

### Plant Material and Growth Conditions

*Arabidopsis* seeds were surface-sterilized with 75% ethanol for 2 min and then dipped in 1% (w/v) NaClO_4_ for 10 min. They were washed with sterilized water six times. Finally, the seeds were kept dipped in the proper volume of sterilized water and incubated in dark at 4°C for 2 days. All *Arabidopsis* seedlings germinated initially on Murashige and Skoog (MS) medium with 1% sucrose, 3.5 mM 2-N-Morpholino ethanesulfonic acid, and 1.2% Agar at pH 5.6 (adjusted with 1 M KOH). Seeds were sown in a horizontal line on plates that were vertically disposed in the growing chamber (16-h light/8-h dark photoperiod) with a light intensity of 200 μmol m^−2^ s^−1^ and 60% relative humidity, at 22°C. Three different Pi level treatments included LP (10 μM), HP (high Pi, 1.25 mM), and Phi (replace Pi with Phi, 1.25 mM). Fifteen-day-old seedlings were photographed. There was a little difference in the vermiculite-hydroponics culture medium. Ten-day-old seedlings germinated on MS medium in the plate were transformed and grown in pots with an equal weight of vermiculite. Four different Pi level MS hydroponics were prepared, which included LP (0.01 mM), HP (1.25 mM), Phi (1.25 mM), and ½Pi + ½Phi (625 μM Pi + 625 μM Phi). An equal volume of the nutrient solution was provided every 3 days. About 35-day-old plants were photographed and their shoot dry weights were measured. The plants continued to grow till the flowering period; about 60-day-old plants were photographed. Until the end of the plant growth period, we collected all the seeds from every single plant. Plant growth conditions were the same as described above.

Seeds of rice (Oryza sativa) cv. ZhongHua 11 were surface-sterilized with 1% nitric acid for 14 h, in dark, and then rinsed with deionized water five times. The seeds germinated at 37°C in the dark for 2 days. Uniformly germinated seeds were cultured hydroponically using Yoshida solution, pH 5.5 (adjusted using 1 M HCl). The solution contained 1.425 mM NH_4_NO_3_, 0.513 mM K_2_SO_4_, 0.998 mM CaCl_2_, 1.643 mM MgSO_4_, 0.25 mM Na_2_SiO_3_, 0.009 mM MnCl_2_, 0.075 μM (NH_4_)_6_Mo_7_O_24_, 0.019 μM H_3_BO_3_, 0.155 μM CuSO_4_, 0.152 μM ZnSO_4_, and 0.125 mM EDTA-Fe (II). The 10-day-old seedlings were transferred to the nutrient solutions with different Pi sources: LP (10 μM), HP (300 μM), Phi (300 μM), LP + Phi (10 μM Pi + 300 μM Phi), and HP + Phi (300 μM Pi + 300 μM Phi). The seedlings were grown in a greenhouse for 20 days, at a 12-h light/12-h dark photoperiod, 32/24°C, and 60% relative humidity, with a photon flux density of 200 µmol m^−2^s^−1^.

### Content Measurements for Pi and Phi

Growth parameters were measured for the WT and transgenic plants under different P conditions. The fresh weights of shoots and roots in the culture medium of LP, HP, and Phi were calculated. The fresh weights and lengths of individual plants in HP, LP + Phi, and HP + Phi culture media were counted. The freshly obtained samples were incubated in a 65°C drying chest for 5 days.

The Pi and Phi concentration was determined as follows: The fully dried sample was ground into powder; 8 ml of deionized water was added to about 50 mg of the dried samples and incubated at 250 rpm for 1 h at 25°C; the sample was placed in a metal bath at 70°C for 2 h and was shaken every 30 min; the sample was centrifuged at 10,000 rpm for 10 min, and 2 ml of the supernatant was filtered using a 0.2-μm filter membrane; the filtrate was measured with an ion chromatograph (Dionex ICS-1100) using appropriate concentrations of NaH_2_PO_4_ and H_3_PO_3_ as standards.

### Statistical Analysis

SPSS Statistics Base software (version 22) was used for statistical analysis. Significant differences were evaluated using one-way ANOVA and Turkey’s test.

## Results

### Fittness of PtxD to Plant

**FIGURE 1:**
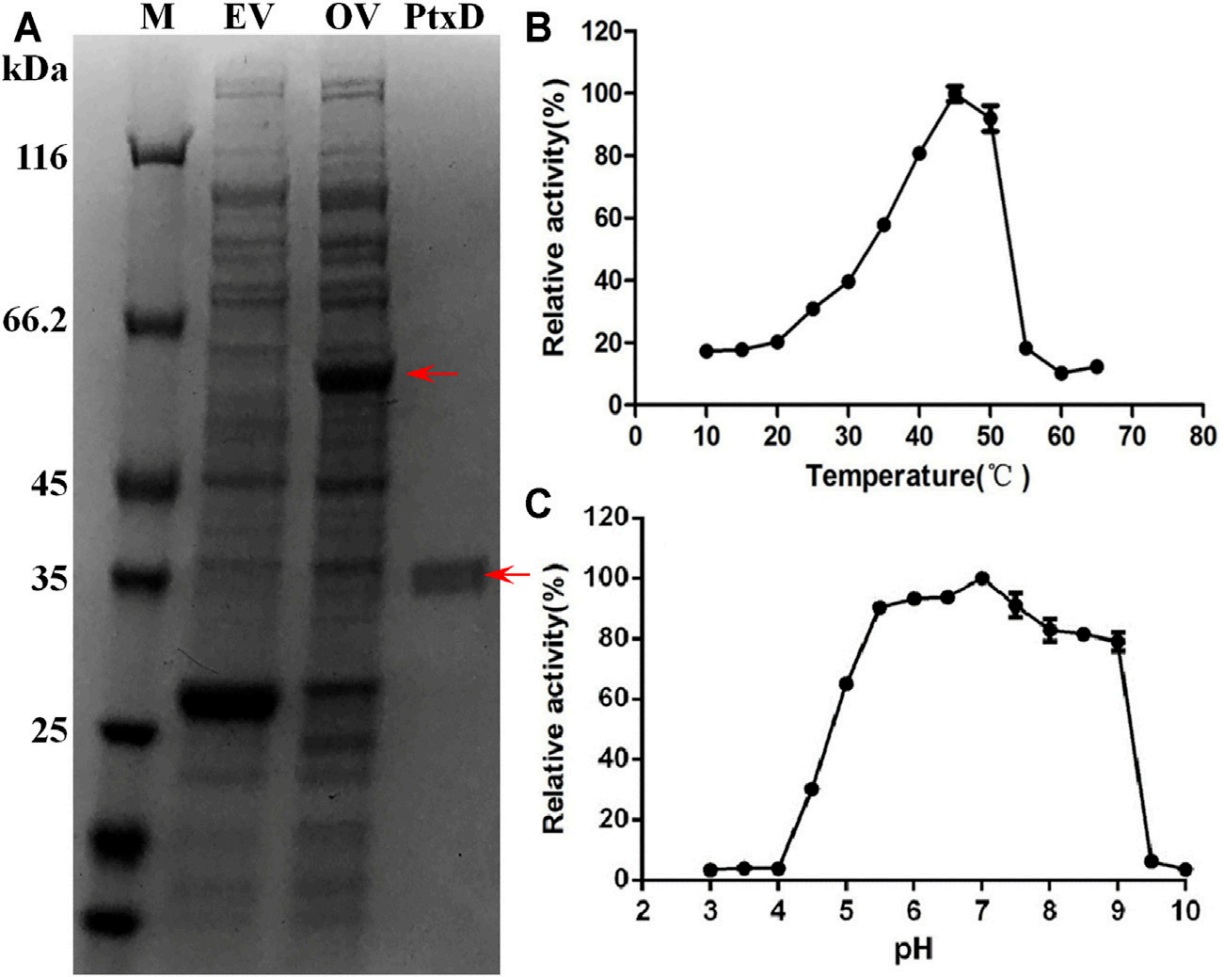
Purification and biochemical properties of PtxD_R4506_. **(A)** Expression and purification of the recombinant PtxD_R4506_ from *E. coli*. The soluble fraction consisting of cellular proteins and purified PtxD were loaded onto SDS-PAGE. M, protein maker; EV, empty vector (pGEX-6P) control; OV, PtxD overexpression vector (pGEX-6P-PtxD); PtxD, purified PtxD_R4506_ protein. **(B)** The effect of temperature on PtxD_R4506_ activity. **(C)** The effect of pH on PtxD_R4506_ activity. All the assay was done in triplicate, and the results were shown as means ± SD.

The *ptxD* gene sequence from *Ralstonia* sp. strain 4,506 was optimized on the basis of the codon bias of *E. coli* and chemically synthesized. For protein PtxD expression in *E. coli*, the synthesized *ptxD*_*R4506*_ gene was cloned into the pGEX-6p-1 expression plasmid. Crude cell extracts were prepared from IPTG-induced strains carrying the *ptxD* overexpression and empty vectors. A band of overexpressed 62-kDa protein was detected in the cell extract from the *ptxD* overexpression sample using SDS-PAGE (Figure 1A). A single protein of approximately 36 kDa was obtained using GST affinity columns, which was consistent with the predicted PtxD protein size from *Ralstonia* sp. strain 4,506 (Figure 1A). The purified PtxD_R4506_ showed maximum Phi oxidation activity at 45°C, and relatively high activity (>90%) was maintained at pH range from 5.5 to 7.5 (Figures 1B,C). The optimal pH of 7.0 rendered PtxD good fittness in plant cell microenvironment. In addition, we tested influence of common ions existing in plant cell including NH_4_^+^, Fe^3+^, Mg^2+^, Mn^2+^, K^+^, Na^+^, and Ca^2+^ on enzyme activity. It was observed that Fe^3+^ could dramatically stimulated the PtxD activity, whereas other ions showed weak enhancing effect, suggesting that these ions commonly existing in plant cell might imposed no inhibition effects on PtxD activity (Supplementary Figure S1).

### Improved Activity of PtxD

To improve the activity of WT PtxD_R4506_, a mutant library was created by introducing random mutations in the *ptxD* gene through error-prone PCR and then screened by 96-well plate assay (Supplementary Figure S2). Using NBT colorimetry, the *ptxD*-M20 mutant that showed the highest PtxD activity was selected from about 10,000 clones. Sequencing analysis showed that the Tyr^139^ of PtxD_R4506_ was substituted by Trp in the improved mutant. To further confirm whether the Tyr^139^ mutation increased the activity of PtxD_R4506_, we purified the mutated PtxD protein and determined its enzymatic kinetic constants. As shown in Table 1, Trp to Tyr mutation at site 139 increased the affinity and turnover number of PtxD protein by approximately 1.5-fold and 2.9-fold higher than WT PtxD, respectively. This indicated that the 139th amino acid residue of PtxD_R4506_ could be used as candidate target for optimizing its catalytic efficiency.

**TABLE 1:** Kinetic constants with phosphite for PtxD_R4506_ and evolved variants.

Enzyme	*K*m (μM)	*k*_cat_ (min^−1^)	*k*_cat_/*K*m (μM^−1^·min^−1^)
PtxD_R450_	9.35 ± 0.37	157.48 ± 1.99	16.84
PtxD_W_ ^a^	6.52 ± 0.34	459.10 ± 6.29	70.46
PtxD_F_ ^b^	5.25 ± 0.58	459.43 ± 12.55	87.54
PtxD_L_ ^b^	11.30 ± 0.60	352.68 ± 7.23	31.21
PtxD_Q_ ^b^	5.51 ± 0.24	541.26 ± 6.40	98.29
PtxD_M_ ^b^	6.78 ± 0.37	399.21 ± 6.12	58.91
PtxD_R_ ^b^	11.94 ± 0.95	158.09 ± 4.27	13.24

Assays were performed in 100 mM Tris-HCl, at 30°C at pH 7.0. The data are represented as means ± SD, of triplicate assays.

a Variant from random mutation library.

b Variants from site-saturation mutation.

To further enhance the enzymatic efficiency of PtxD, saturation mutagenesis was performed by introducing 18 different amino acids excluding Trp and Tyr, at the 139th site of PtxD using overlap extension PCR. The activity of these 18 mutants was screened by using the NBT assay, which was employed for random mutation libraries screening. As compared to the WT, six mutants retained comparable or even higher enzymatic activity; whereas the other 13 mutants showed no obvious improvement in activity (Supplementary Figure S3). The six PtxD variants were purified to high homogeneity by GST affinity chromatography. Enzyme kinetic constants were measured and compared with the parent enzyme. We found that four variants (Y139F, Y139Q, Y139M, and Y139L) obtained from saturation mutagenesis exhibited significantly enhanced PtxD activity, whereas one variant (Y139R) had a slightly reduced PtxD activity (Table 1). Notably, among all the tested PtxD proteins, the variant Y139Q showed the lowest K_m_ and highest *k*_cat_ with phosphite, with an enhanced 5.8-fold catalytic efficiency as compared to the WT PtxD (Table 1).

### *Arabidopsis* can use Phi as Sole Phosphorus Source by *ptxD*_*Q*_ Overexpression

**FIGURE 2:**
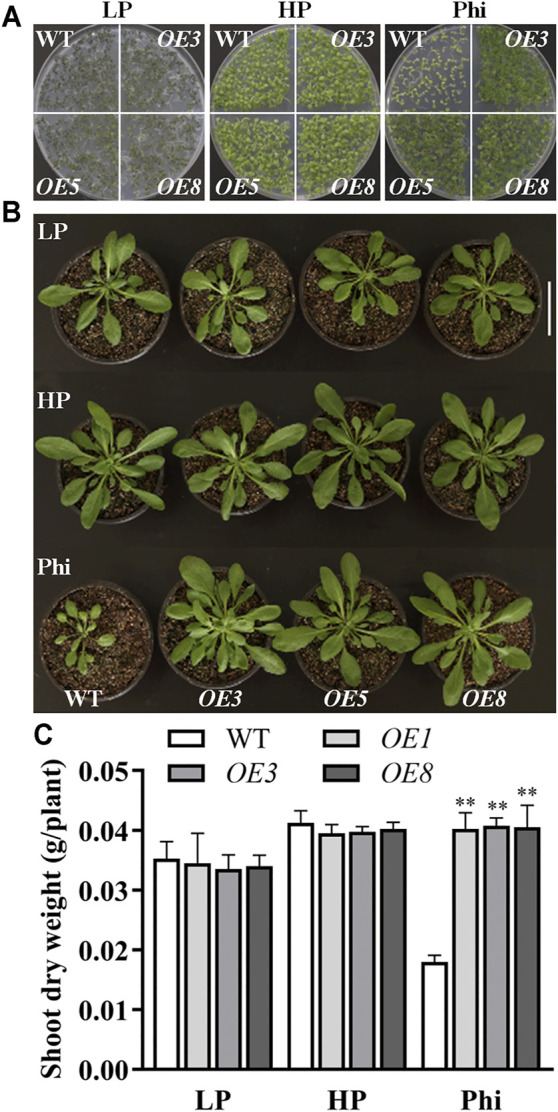
Phenotypes of *ptxD*_*Q*_ transgenic *Arabidopsis* with different P sources. **(A)** Comparative growth of seedlings from *ptxD*_*Q*_ overexpression lines (*OE3*, *OE5*, and *OE8*) and WT (*Col-0*) grown on MS plate medium containing 5 μM phosphate (LP), 1.25 mM phosphate (HP), or 1.25 mM phosphite (Phi). **(B)** Transgenic and WT *Arabidopsis* were grown in a sterilized mixture of sphagnum peat moss/perlite soil with MS solution containing 5 μM phosphate (LP), 1.25 mM phosphate (HP), or 1.25 mM phosphite (Phi). Scale bar = 5 cm. **(C)** Shoot dry weight of seedlings from WT and *PtxD*_*Q*_ overexpression *Arabidopsis* grown in a sterilized mixture of sphagnum peat moss/perlite soil mix. The data are shown as means ± SD for four replicates. Asterisks indicate significant differences as compared with WT plants (Turkey’s test, **p ≤ 0.01).

To overexpress the *ptxD*_*Q*_ in *Arabidopsis*, the *ptxD*_*Q*_ sequence was optimized for the codon bias of plants and chemically synthesized (Supplementary Figure S4). The synthetic *ptxD*_*Q*_ gene was cloned into the plant transformation vector pTF101-Ubi. By Agrobacterium-mediated transformation, 12 independent *ptxD*_*Q*_ overexpression *Arabidopsis* lines were generated (Supplementary Figure S5). The homozygous T3 seeds germinated and were grown on MS medium with LP, HP, and Phi. The *ptxD*_*Q*_ overexpression lines showed similar growth phenotypes as the WT under both LP and HP conditions. Interestingly, although the non-transgenic *Arabidopsis* did not grow on medium with phosphite as the sole source of P, the *ptxD*_*Q*_ overexpression lines could grow normally under Phi treatment condition (Figure 2A). To further examine the ability of the *ptxD*_*Q*_ overexpression lines to utilize Phi as the sole source of P, 10-day-old seedlings were transformed and grown in pots with an equal weight of vermiculite. Nutrient solutions containing LP, HP, and Phi were used to water the plants regularly. The growth of seedlings with *ptxD*_*Q*_ overexpression was similar to Col-0 under both Pi-deficient and Pi-sufficient conditions (Figures 2B,C). Although the growth of Col-0 seedlings was completely inhibited by Phi treatment, the *ptxD*_*Q*_ overexpression grew well under Phi treatment condition (Figures 2B,C).

**FIGURE 3:**
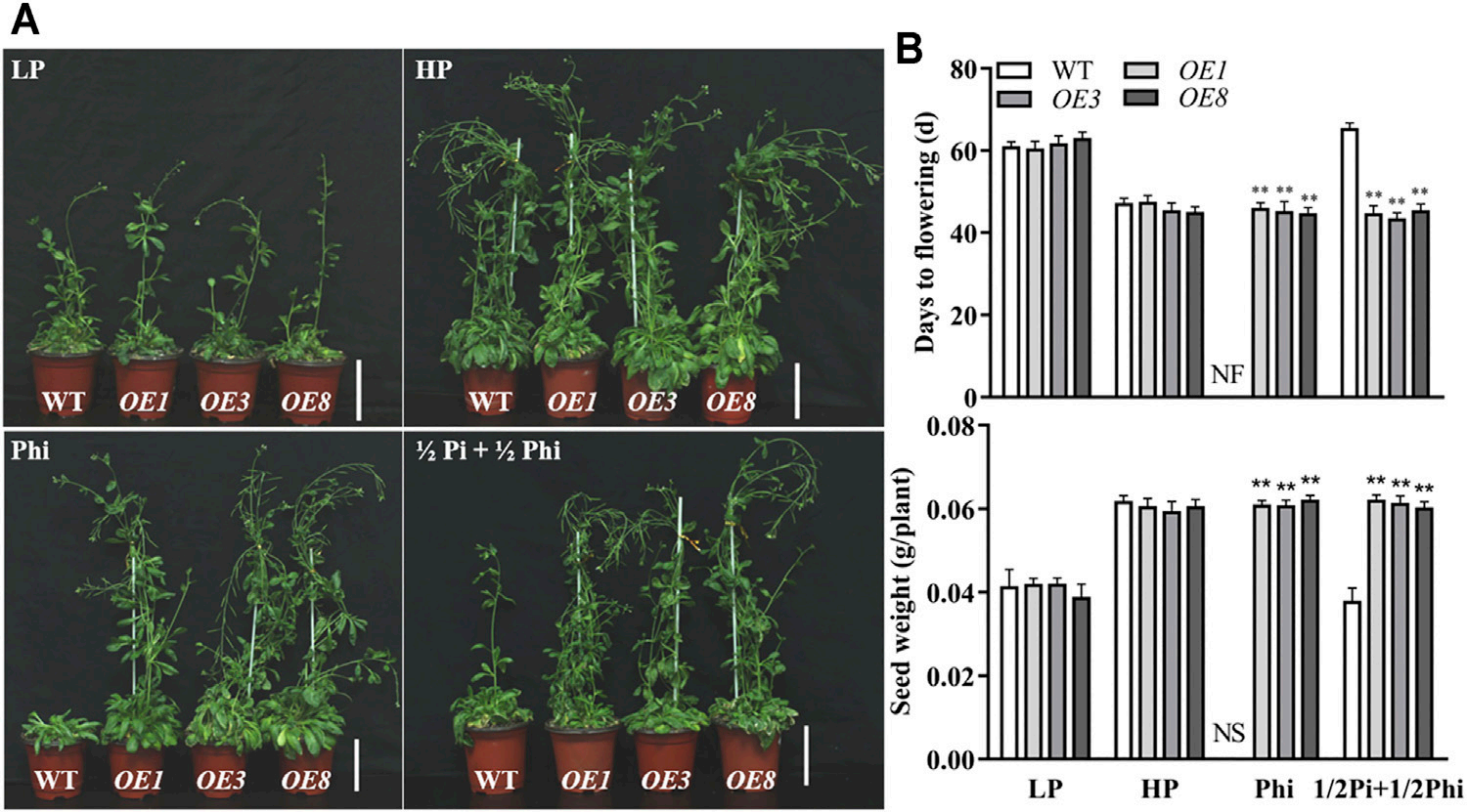
Effect of different phosphate- and phosphite-based nutrition on flowering time and yield in WT and *ptxD*_*Q*_ transgenic *Arabidopsis*. **(A)** Phenotypes of WT (*Col-0*) and transgenic (*OE3*, *OE5*, and *OE8*) plants at seed setting stage under different phosphorus nutrition conditions. Scale bar = 2 cm. **(B)** Days for flowering time and seed yield of WT and transgenic plants. Transgenic and WT *Arabidopsis* were grown in a sterilized mixture of sphagnum peat moss/perlite soil mix fertilized with MS solution containing 5 μM phosphate (LP), 1.25 mM phosphate (HP), 1.25 mM phosphite (Phi), or ½Pi + ½Phi (625 μM Pi + 625 μM Phi). The data are shown as means ± SD for five replicates. Asterisks indicate significant differences as compared with WT plants (Turkey’s test, **p ≤ 0.01). NF, no flower; NS, no seed.

We further examined the effects of different P nutrient conditions on the agronomic traits in *Arabidopsis*. As expected, LP treatment delayed flowering time and produced fewer seeds as compared to the HP treatment condition. The flowering time and seed production were similar between WT plants and *ptxD*_*Q*_ overexpression lines under both Pi-deficient and Pi-sufficient conditions (Figure 3). With phosphite as the only P source, flowering was inhibited and no seeds were produced in WT plants (Figure 3). In contrast, the flowering time and seed production of *ptxD*_*Q*_ overexpression plants were not affected by Phi treatment. This indicated that the *ptxD*_*Q*_ overexpression plants could use phosphite as the only P source to complete their life cycle (Figure 3). We further treated the plants with a combination of phosphate and phosphite in the ratio of 1:1. Interestingly, the WT plants showed a delayed flowering time and reduced seed production similar to the Pi-deficient condition; the *ptxD*_*Q*_ overexpression plants showed a similar flowering time and seed production as in Pi-sufficient conditions (Figure 3).

### Phosphite Stimulates the Growth of *ptxD*_*Q*_ Overexpression Rice Plants Under Both LP and HP Conditions

**FIGURE 4:**
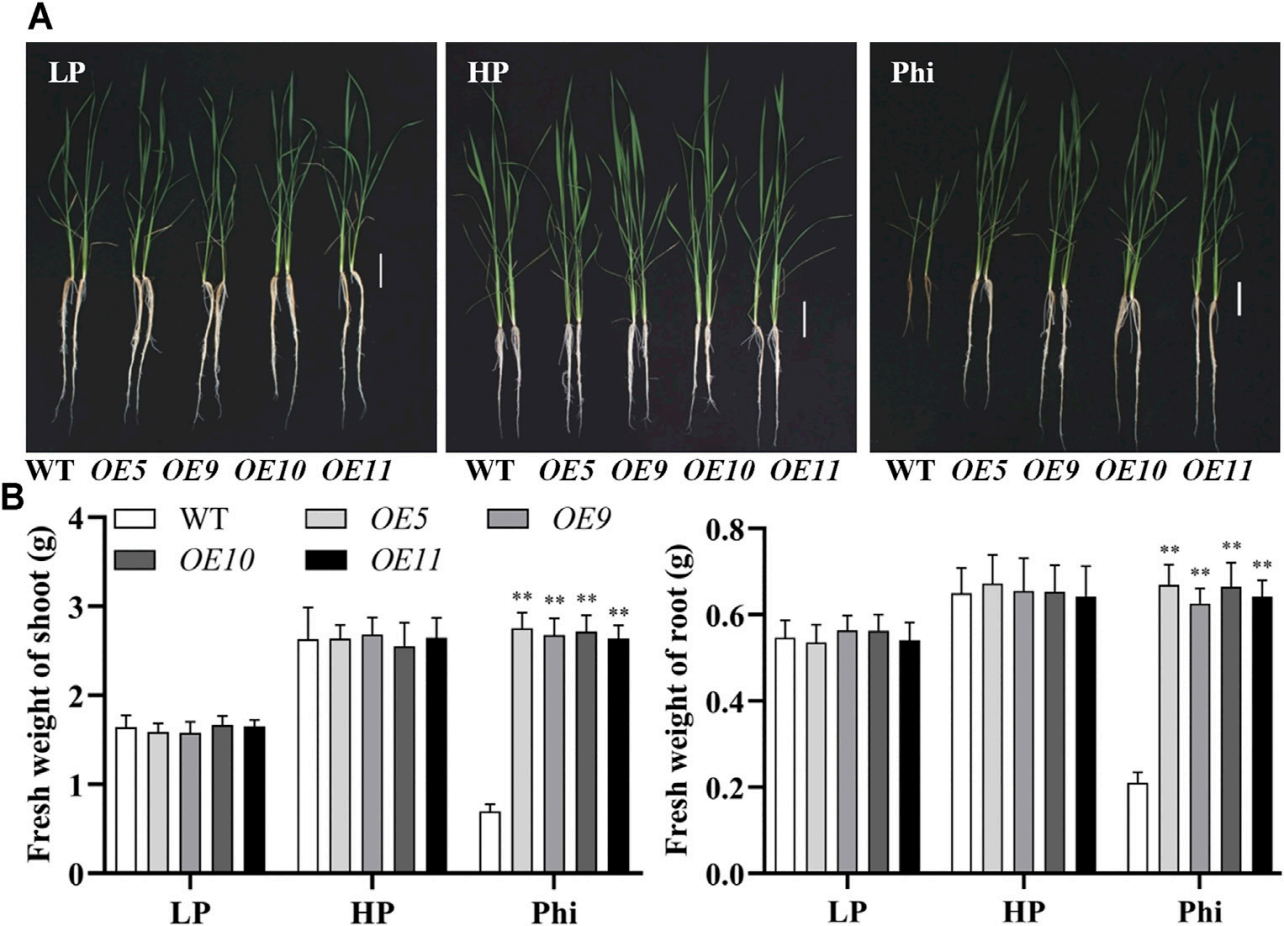
Growth performance **(A)** and fresh weight **(B)** of WT (*ZH11*) and *ptxD*_*Q*_ overexpression (*OE5*, *OE9*, *OE10*, and *OE11*) rice with different P sources. Fourteen-day-old seedlings were transferred to nutrient solutions containing 10 μM phosphate (LP), 300 μM phosphate (HP), or 300 mM phosphite (Phi). Scale bar = 5 cm. The data are shown as means ± SD for five replicates. Asterisks indicate significant differences as compared with WT plants (Turkey’s test, **p ≤ 0.01).

**FIGURE 5:**
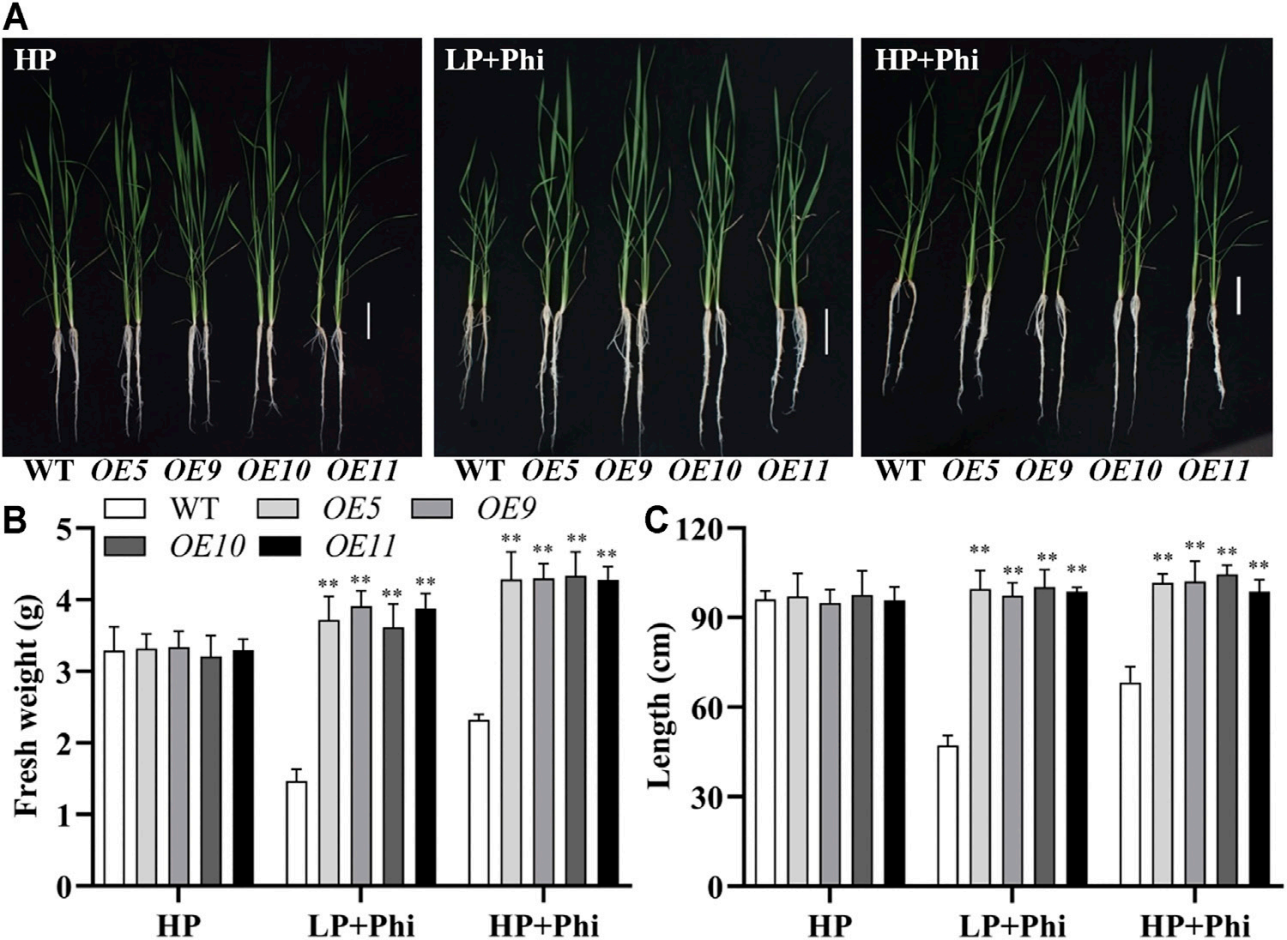
Effect of phosphite on the growth of WT and *ptxD*_*Q*_ transgenic rice under low and high phosphate conditions. Growth performance **(A)**, fresh weight **(B)**, and plant height **(C)** of WT (*ZH11*) and *PtxD*_*Q*_ overexpression (*OE5*, *OE9*, *OE10*, and *OE11*) rice grown under different phosphorus nutrient conditions. Fourteen-day-old seedlings were transferred to nutrient solutions containing 300 μM phosphate (HP), 10 μM phosphate plus 300 μM phosphite (LP + Phi), or 300 mM phosphite plus 300 μM phosphite (HP + Phi). Scale bar = 5 cm. The data are shown as means ± SD for five replicates. Asterisks indicate significant differences as compared with WT plants (Turkey’s test, **p ≤ 0.01).

In addition to *Arabidopsis*, the *ptxD*_*Q*_ gene was also transformed into the rice, and 11 independent overexpression lines were generated (Supplementary Figure S6). To examine whether the *ptxD*_*Q*_ overexpression rice could utilize Phi as the sole source of P, we performed hydroponic experiments with T_2_ generation transgenic lines. The transgenic plants showed similar growth as WT plants under both Pi-deficient (LP) and Pi-sufficient (HP) conditions. When Phi was provided as the sole source of P in the nutrient solution, the WT plants were smaller than those under LP conditions. Under a prolonged Phi treatment, the WT seedlings died (data not shown). In contrast, when Phi was the sole source of P, the *ptxD*_*Q*_ overexpression plants grew normally as in the Pi-sufficient conditions (Figure 4). We compared the effects of Phi on the growth of transgenic rice under LP and HP conditions. Although Phi treatment inhibited the growth of non-transgenic plants under both LP and HP conditions, the *ptxD*_*Q*_ overexpression rice had similar plant heights as in the HP conditions under Phi treatment. Moreover, the fresh weight of *ptxD*_*Q*_ overexpression rice treated with Phi increased by approximately 14.9% and 30.7%, under LP and HP conditions, respectively, as compared to the plants grown under HP conditions (Figure 5). Together, these results indicated that overexpression of *ptxD*_*Q*_ conferred the ability to use Phi as a P resource in rice plants, which could further stimulate the growth of transgenic plants under both LP and HP conditions. We further tested the effect of spaying Phi on the growth of rice under Pi-sufficient condition. Spaying high concentration of Phi significantly inhibited the growth of WT plants (Supplementary Figure S7). Interesting, *ptxD*_*Q*_ overexpression rice plants are resistance to spaying Phi on the shoots (Supplementary Figure S7).

### Overexpression of *ptxD*_*Q*_ Altered the Phosphite Uptake Capacity in Rice

**FIGURE 6:**
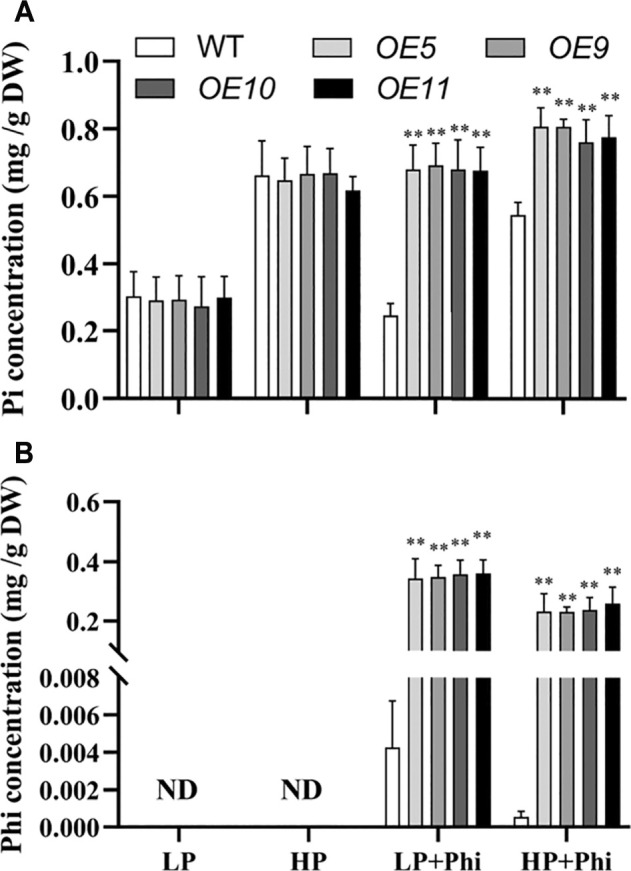
Pi **(A)** and Phi **(B)** concentrations of WT (*ZH11*) and *ptxD*_*Q*_ (*OE5*, *OE9*, *OE10*, and *OE11*) transgenic rice grown under different phosphorus nutrient conditions. Fourteen-day-old seedlings were transferred to nutrient solutions containing 10 μM phosphate (LP), 300 μM phosphate (HP), 10 μM phosphate plus 300 μM phosphite (LP + Phi), or 300 mM phosphite plus 300 μM phosphite (HP + Phi). The data are shown as means ± SD for five replicates. Asterisks indicate significant differences as compared with WT plants (Turkey’s test, **p ≤ 0.01).

The Pi and Phi contents under different P treatment conditions were measured in the WT and transgenic plants. The Pi content showed no significant differences between WT and transgenic plants under LP and HP conditions. The addition of Phi in the nutrient solutions with LP and HP decreased the Pi content by 19.3% and 17.8%, respectively, in the shoots of WT plants (Figure 6A). However, Pi content in the shoots of transgenic plants increased by approximately 135% and 21.1% under Phi treatment for LP and HP conditions, respectively (Figure 6A). We could not detect any Phi in the WT and transgenic plants under LP and HP conditions without Phi applicant (Figure 6B). When Phi was added to the nutrient solution, its content in WT and transgenic plants was higher under the LP condition as compared to the HP condition. However, Phi content in WT was very low compared with that in transgenic plants when Phi was added into the nutrient solution under both LP and HP conditions (Figure 6B).

## Discussion

Globally, the scarcity of available P in soil is a big limitation for agriculture productivity (Shen et al., 2011). Exceeded application of Pi fertilizers for crop growth continues due to the low efficiency of utilization of the Pi fertilizers. Runoff of the Pi fertilizers not only increases the cost of agriculture but is also a major pollutant in most surface waters. Phi-based fertilizers as compared to Pi-based fertilizers may reduce 30%–50% P fertilizer requirements and eutrophication in water (Achary et al., 2017). However, plants cannot metabolize Phi, despite their effective uptake of Phi by Pi transporters (Thao and Yamakawa, 2009). Interestingly, engineered plants with *ptxD* genes can grow with Phi as the sole P fertilizer (López-Arredondo and Herrera-Estrella, 2012; Manna et al., 2016). The *ptxD*_*PS*_ from *Pseudomonas stutzeri* WM88 is the most widely used PtxD gene in transgenic plants, but overexpression of *ptxD*_*PS*_ only showed a significant effect for Phi utilizing efficiency under no or LP conditions (López-Arredondo and Herrera-Estrella, 2012; López-Arredondo and Herrera-Estrella, 2013; Loera-Quezada et al., 2016; Manna et al., 2016; Nahampun et al., 2016; Pandeya et al., 2018). In this study, we have explored to screen high efficiency PtxDs and its application in plant engineering.

It is reported that 10%–67% of total culturable bacteria can utilize Phi as a sole P source in natural aquatic and terrestrial environments (Figueroa and Coates, 2017). In addition to *ptxDps*, several *ptxD* genes have been cloned from different bacterial strains including *Ralstonia* sp.4506, *Nostoc* sp, and *Methylobacterium extorquens* AM1 (Andrea and William, 2007; Hirota et al., 2012). Interestingly, the *ptxD*_*R4506*_ from *Ralstonia* sp. strain 4506 with only 54.9% identity of *ptxD*_*PS*_ showed more than 6.7-fold catalytic efficiency as compared to *ptxD*_*PS*_ (Hirota et al., 2012). In this study, we screened highly efficient PtxD using the directed evolution of PtxD_R4506_. A mutant strain (*ptxD*-M20) with enhanced PtxD activity was obtained. PtxD protein of the mutant strain had a single mutation at the 139th site (Y to W) as compared to the WT PtxD_R4506_. We additionally introduced other 18 amino acids into at 139th site of PtxD_R4506_. Interestingly, only four of the 18 mutant strains showed significantly enhanced PtxD activity (Supplementary Figure S3, Table 1), which indicated the critical function of the 139th site for phosphite binding or oxidizing potential. X-ray crystallographic analysis revealed that PtxD has two catalytic domains (Liu et al., 2019; Liu et al., 2021). Molecular modeling of the PtxD_R4506_ protein shows that the 139th tyrosine of PtxD_R4506_ locates in a random coil connecting two catalytic domains (Supplementary Figure S8A). The side chain of tyrosine may influence interaction of the two domains by transverse between them (Supplementary Figure S8B). When the 139th tyrosine mutated to glutamine or phenylalanine, it may increase stability of the two catalytic domains, resulting in an enhanced PtxD activity (Supplementary Figure S8). It is noteworthy that mutation of the 139th tyrosine to other amino acids only changed the distance and position of the two domains, but the basic structures of mutated PtxD were unchanged. Although substitution of the 139th tyrosine with other amino acids influenced PtxD activity, it could not eliminate its activity by any amino acids (Supplementary Figure S3).

Among the selected variants, the PtxD_Q_ had the highest catalytic efficiency and showed 5.83-fold increased catalytic efficiency than PtxD_R4506_ and was further used for transforming *Arabidopsis* and rice. As expected, the transgenic plants showed normal growth when Phi was supplied as the sole P source. One of the purposes of Phi-based fertilization is to establish a weed control system, which cannot be achieved under a high phosphate condition (Heuer et al., 2017). Interestingly, Phi treatment not only stimulated the growth of the *ptxD*_*Q*_ overexpressing plants under the LP condition but also increased their growth under the HP condition (Figure 5). This may be attributed to the higher catalytic efficiency of *ptxD*_*Q*_ than *ptxD*_*PS*_. We found that spaying high concentration of Phi solutions inhibited the growth of WT plants even under HP supply. In contrast, the growth of transgenic plants was not affected by spaying Phi (Supplementary Figure S7). Furthermore, both the *ptxD*_*Q*_ transgenic *Arabidopsis* and rice showed normal growth without any yield compromise. Thus, screening highly efficient PtxD to transform plants is a potential technological application for Phi fertilizer and weed control in real field conditions.

When the *Arabidopsis* were supplied with 625 μM Pi and 625 μM Phi in the soil, the growth of the WT *Arabidopsis* (*col-0*) was similar to those treated with 5 μM Pi (Figure 3). When the rice was treated with 300 μM Pi and 300 μM Phi (HP + Phi), the fresh weight and length of the seedlings were significantly lower than those in the 10 μM Pi treatment condition (LP) (Figure 5). These phenomena were as previously reported results where Phi competitively suppressed the Pi uptake and affected the plant metabolism (Costas et al., 2001; Thao and Yamakawa, 2009). As expected, Pi content in WT seedlings that grow under LP + Phi or HP + Phi was lower than those treated with LP or HP without Phi, respectively (Figure 6). Although Phi was detected in WT under Phi treatment conditions, its content was extremely low in WT seedlings as compared to the transgenic plants (Figure 6). However, Phi content in transgenic plants was relatively high, which indicated the amount of Phi stored in the plants. Thus, the xenobiotic Phi may be extruded out of the cells in WT plants (Danova-Alt et al., 2008). The metabolic and Pi states of the cells have a strong influence on the subcellular localization of Phi. Pi starved cells predominantly accumulate Phi in the cytoplasm and do not store Phi in the vacuole, whereas Pi-preloaded cells import Phi into vacuole (Danova-Alt et al., 2008). Overexpression of *ptxD*_*Q*_ conferred the metabolizing ability of Phi in transgenic plants, which mimic the Pi-preloaded cells. Therefore, the transgenic plants favored the storage of Phi in vacuole other than extrude the Phi out of cells.

## Data Availability

The original contributions presented in the study are included in the article/Supplementary Material; further inquiries can be directed to the corresponding authors.
