# Supplementary material for: Improved the Activity of Phosphite Dehydrogenase and its Application in Plant Biotechnology
Source: Front Bioeng Biotechnol. 2021 Nov 25;9:764188. doi: 10.3389/fbioe.2021.764188 (PMC8655118; doi:10.3389/fbioe.2021.764188)
Supplement: Supplementary file 1 [file DataSheet1.PDF]

Figure S1

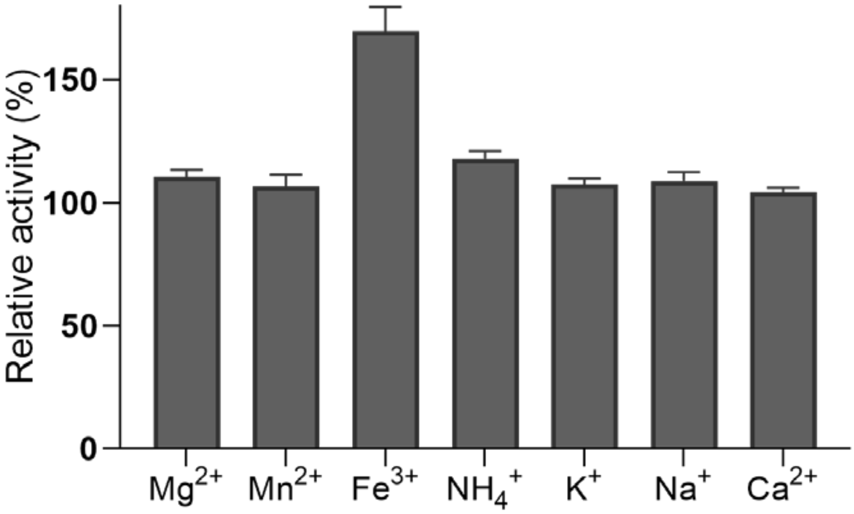

Figure S1. The effect of ions on PtxDR4506 activity. The relative activity in the presence of NH_4^+, Fe^{3+},Mg^{2+}, Mn^{2+},K^+,Na^+ and Ca^{2+} was determined via standard enzyme assay described in the methods.

Figure S2

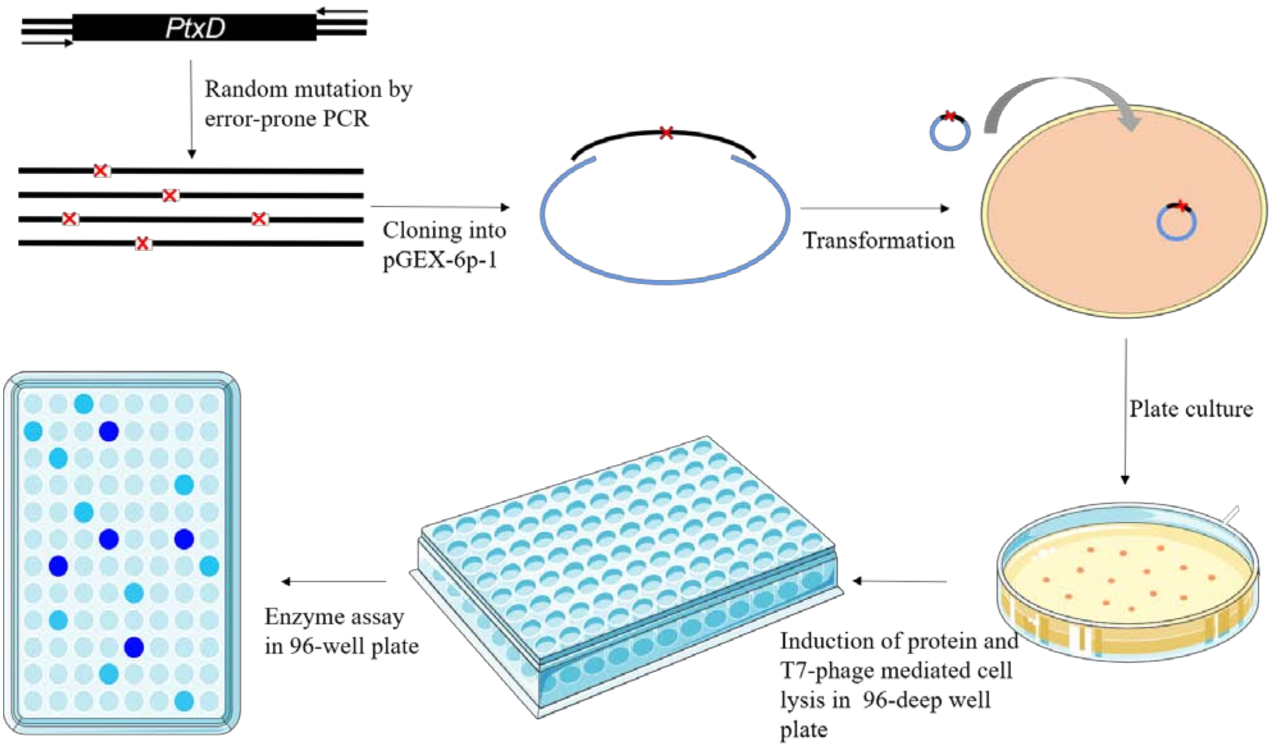

Figure S2. Scheme of the mutation library construction and high-through screening.

Figure S3

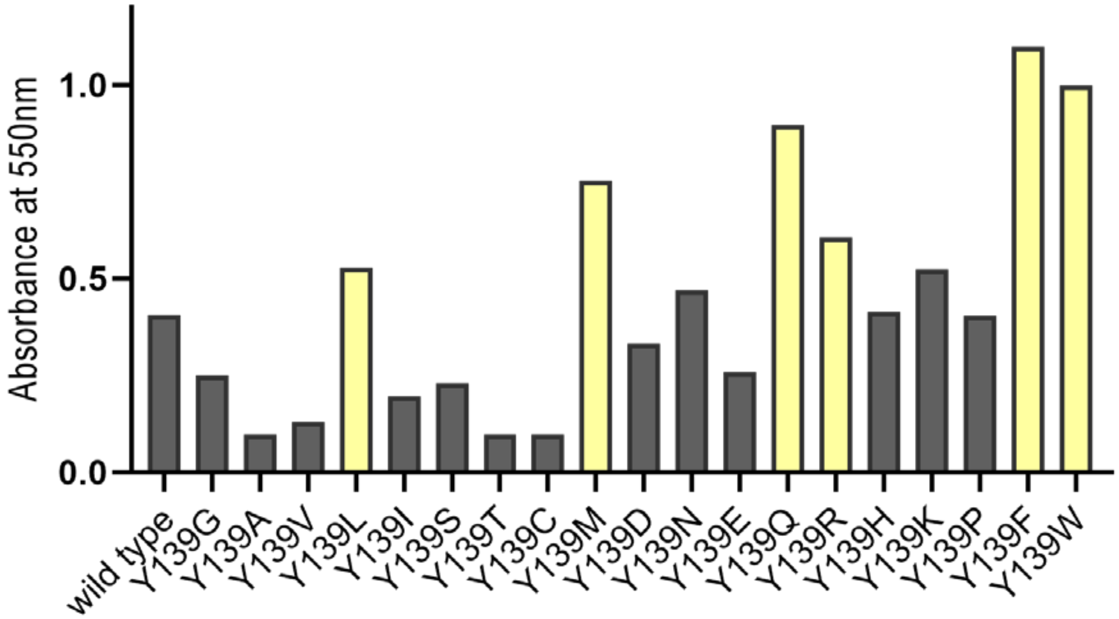

Figure S3. Screening of site-saturation mutagenesis. The cell lysates containing ptxD variants were assayed by determing the absorbance at 550nm.

Figure S4

➢ **Protein sequence of PtxD$_Q$:**

MKPKVVLTHWVHPEIIELLSASADVIPNTTRETLPRSEVIARAKDADALMAFMPDSIDS
AFLEECPKLRVIGAALKGYDNFDVNACTRHGVWLTIVPDLLTIPTAELTIGLLLGLTRHM
LEGDRQIRSGHFQGWRPTLQGSGLTGKTLGIIGMGAVGRAIAQRLAGFEMNLLYCDPIP
LNAEQEKAWHVQRVTLDELLEKCDYVVPMVPMAAETLHLIDATALAKMKTGSYLINA
CRGSVVDENAVIAALASGKLAGYAADVFEMEEWIRADRPQAIPKALLDNTAQTFFTPH
LGSAVKEVRLEIERQAAMNIIQALAGEKPMGAINQPYPGVKAA

➢ **Codon-optimized sequence of *ptxD$_Q$*:**

ATGAAGCCGAAGGTGGTCCTCACCCACTGGGTGCACCCGGAGATCATCGAGCTCCT
CAGCGCCTCCGCCGACGTGATCCCGAATACCACGCGCGAGACGCTCCCACGCTCCG
AAGTCATTGCCAGGGCCAAGGACGCCGATGCGCTGATGGCGTTCATGCCGGACAGC
ATCGATAGCGCGTTCCTCGAGGAGTGTCCGAAGCTCAGGGTCATCGGCGCCGCCCTG
AAGGGCTACGACAACTTCGACGTGAACGCGTGCACCAGGCATGGCGTCTGGCTCAC
CATTGTGCCGGATCTCCTCACCATCCCGACCGCCGAGCTCACGATTGGCCTGCTCCT
GGGCCTCACCAGGCACATGCTGGAAGGCGACAGGCAGATCCGCTCCGGCCACTTCC
AGGGCTGGAGGCCAACCCTCCAAGGCAGCGGCCTCACCGGCAAAACCCTGGGCATT
ATTGGCATGGGCGCCGTGGGCAGGGCCATCGCCCAGCGCCTGGCGGGCTTCGAGAT
GAACCTCCTCTACTGCGACCCGATCCCACTCAACGCCGAGCAGGAGAAGGCCTGGC
ACGTGCAGAGGGTCACCCTCGACGAGCTGCTGGAGAAGTGCGACTACGTGGTCCCA
ATGGTGCCGATGGCCGCGGAGACCCTCCACCTGATTGACGCCACCGCCCTCGCCAA
GATGAAGACCGGCTCCTACCTCATCAACGCCTGTCGCGGGAGCGTGGTGGATGAGA
ACGCGGTCATTGCCGCGCTCGCCAGCGGCAAACTCGCCGGCTATGCCGCGGACGTG
TTCGAGATGGAAGAGTGGATCCGCGCGGATAGGCCACAGGCCATCCCGAAAGCCCT
CCTCGACAACACGGCCCAGACCTTCTTCACCCCGCATCTGGGCTCCGCCGTGAAGG
AGGTGAGGCTCGAGATTGAGAGGCAAGCCGCCATGAACATCATTCAGGCCCTCGCG
GGCGAGAAGCCGATGGGGGCCATCAACCAGCCGTACCCAGGCGTGAAGGCCGCC

Figure S5

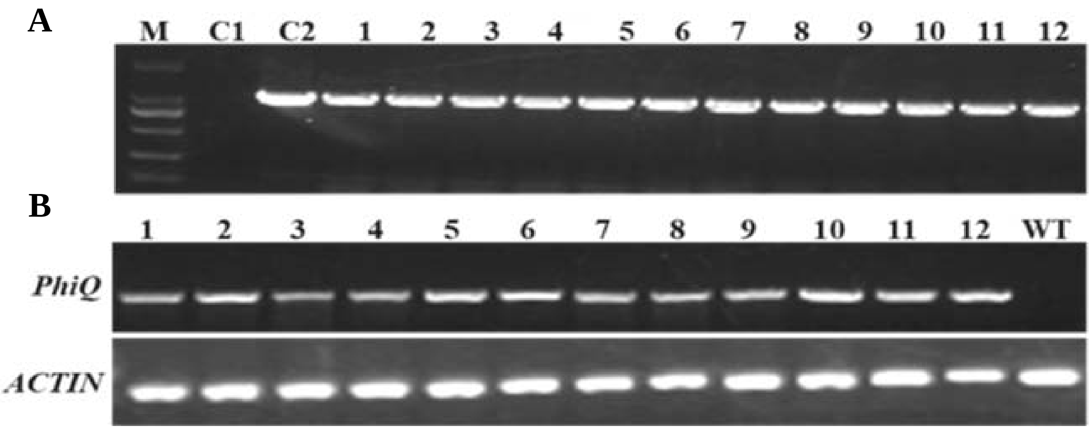

Figure S5. (A) PCR-based detection of independent transgenic lines in T1 generation *Arabidopsis*. The extracted DNA of each transgenic line was used as a template for PCR-based detection of $ptxD_{Q}$. M: DL 2000 DNA Mark; C1: WT control; C2: Positive control. Lanes 1 to 12 were independent *Arabidopsis* transgenic lines. **(B) RT-PCR analysis of $ptxD_{Q}$ expression in transgenic independent lines.** The cDNA from each transgenic line was used as a template to perform RT-PCR-based detection of $ptxD_{Q}$. The actin gene was used as the internal control and WT was the negative control.

Figure S6

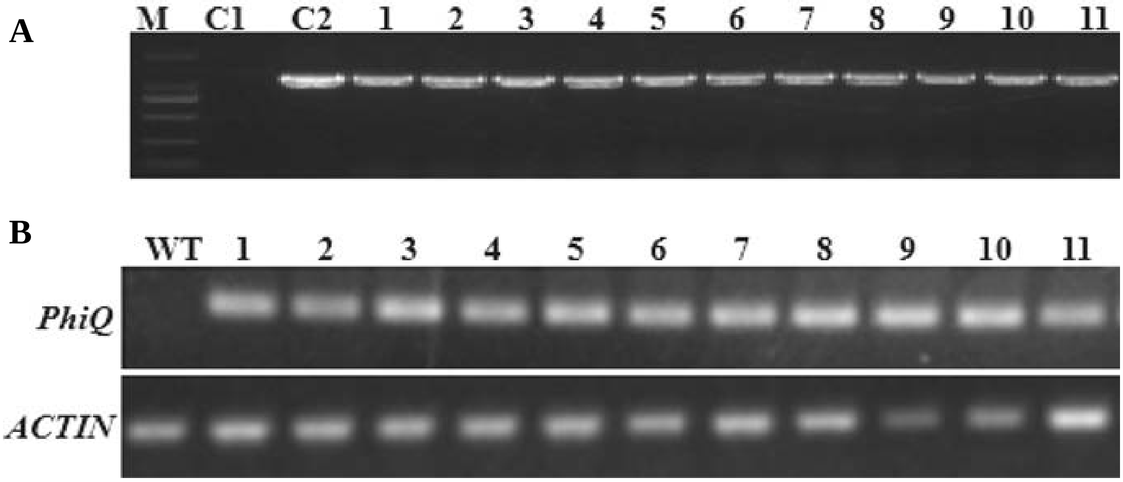

Figure S6. (A) PCR-based detection of independent transgenic lines in T1 generation rice. The extracted DNA from each transgenic line was used as a template for PCR-based detection of $ptxD_{Q}$. M: DL 2000 DNA Mark; C1: WT control; C2: Positive control. Lanes 1 to 11 were independent rice transgenic lines.

(B) RT-PCR analysis of *ptxD*$_{Q}$ expression in transgenic independent lines. The cDNA from each transgenic line was used as a template to perform RT-PCR-based detection of $ptxD_{Q}$. The ACTIN gene was used as the internal control and the WT sample as a negative control.

Figure S7

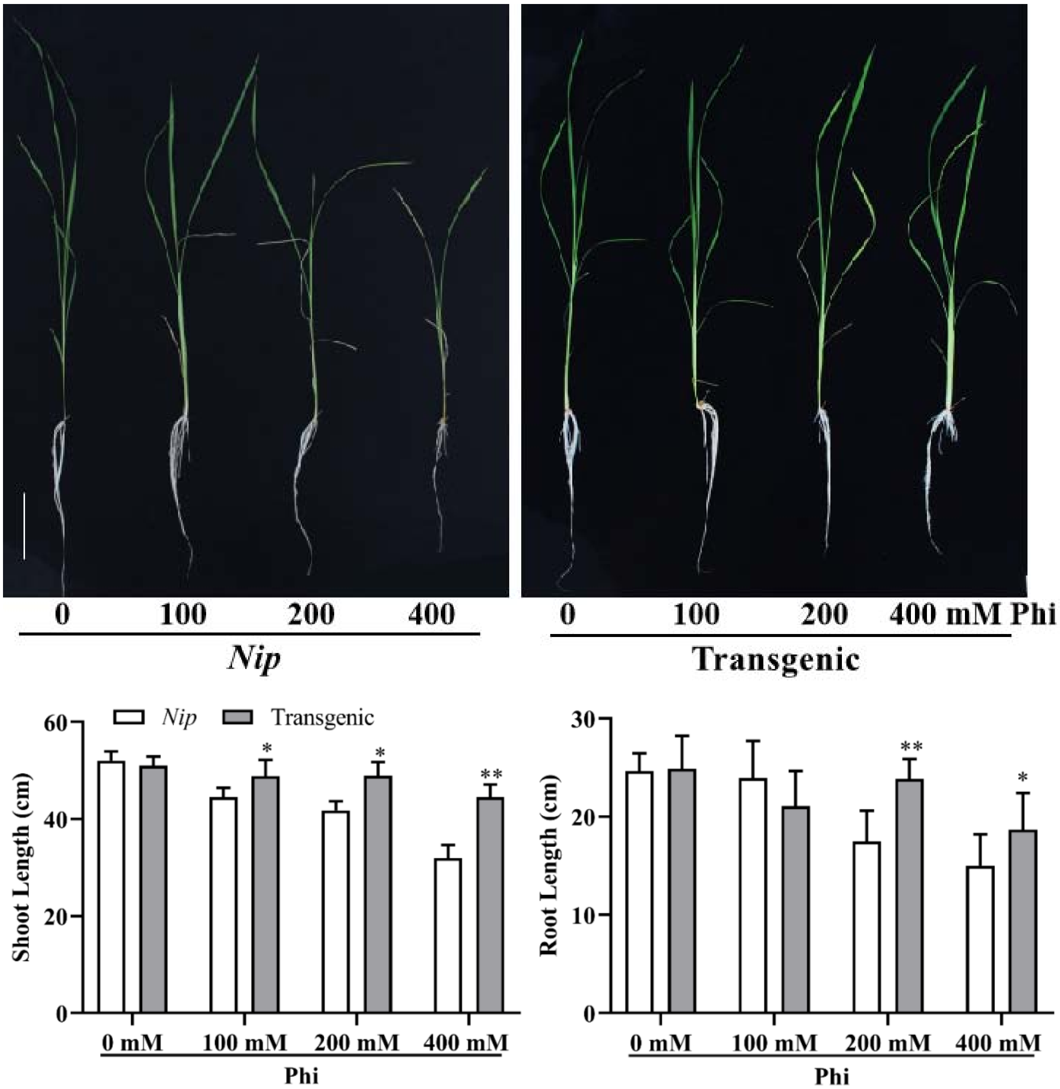

Figure S7. Phenotypes of WT (*Nip*) and *ptxD*$_Q$ transgenic rice spaying with different concentration of Phi. 14-day-old seedlings were transferred to nutrient solutions containing 300 μM phosphate and sprayed with Phi. Scale bar = 5 cm. The data are shown as means ± SD for five replicates. Asterisks indicate significant differences as compared with WT plants (Turkey's test, ** $P \leq 0.01$).

Figure S8

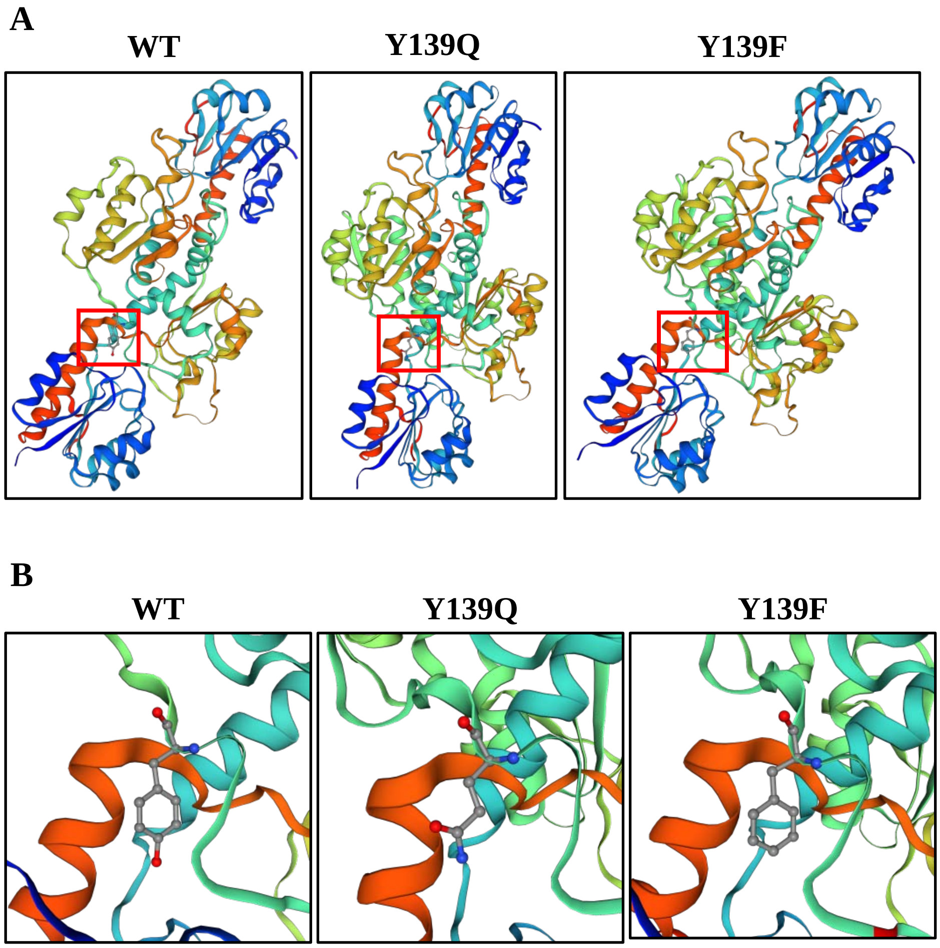

Figure S8. Simulate the three-dimensional protein structure of the PtxD mutant. The high-reliable Swiss-modle was used to simulate the three-dimensional structure of $PtxD_Q$ and other variants. The 139th position was mutated to a different amino acid and showed a different chemical structure.
